# Fatty acid‐binding protein 5 function in hepatocellular carcinoma through induction of epithelial–mesenchymal transition

**DOI:** 10.1002/cam4.1020

**Published:** 2017-04-04

**Authors:** Takanori Ohata, Hideki Yokoo, Toshiya Kamiyama, Moto Fukai, Takeshi Aiyama, Yutaka Hatanaka, Kanako Hatanaka, Kenji Wakayama, Tatsuya Orimo, Tatsuhiko Kakisaka, Nozomi Kobayashi, Yoshihiro Matsuno, Akinobu Taketomi

**Affiliations:** ^1^ Department of Gastroenterological Surgery IHokkaido University Graduate School of MedicineSapporoJapan; ^2^ Department of Surgical PathologyHokkaido University HospitalSapporoJapan

**Keywords:** Epithelial‐mesenchymal transition, fatty acid‐binding protein, hepatocellular carcinoma, metastasis, prognosis

## Abstract

Hepatocellular carcinoma (HCC) is a highly prevalent cancer with poor prognosis. The correlation between overexpression of fatty acid‐binding protein 5 (FABP5) and malignant potential of tumor growth and metastasis in several cancers has been previously reported. However, the correlation between FABP5 expression and HCC malignant behavior remains unknown. We compared FABP5 expression and patient characteristics in paired HCC and adjacent noncancerous liver tissues from 243 patients who underwent surgical resection of primary HCC. Cell proliferation, invasion, and migration assays were performed in HCC cell lines overexpressing FABP5 or downregulated for FABP5. Tumor growths were monitored in xenograft model, and liver and lung metastasis models were established. In the 243 HCC patients, FABP5‐positive staining (*n* = 139/243, 57.2%) was associated with poor prognosis and recurrence (*P* < 0.0001) and showed positive correlation with distant metastasis, tumor size and vascular invasion (*P* < 0.05). Cell proliferation, invasion, and migration in vitro were enhanced by upregulation of FABP5 and decreased by downregulation of FABP5 in HCC cell lines. Similar results in tumor formation and metastasis were obtained through in vivo analyses. PCR array results revealed upregulation of SNAI1 in FABP5‐overexpressing HepG2 cells. Western blot analysis showed significantly increased expression of E‐cadherin and ZO‐1 and decreased SNAI1 expression and nuclear translocation of *β*‐catenin by knockdown of FABP5. We revealed a significant role for FABP5 in HCC progression and metastasis through the induction of epithelial‐to‐mesenchymal transition. FABP5 may be a potential novel prognostic biomarker and new therapeutic target for HCC.

## Introduction

Hepatocellular carcinoma (HCC) is a highly prevalent cancer and the third cause of cancer‐related death worldwide 1. Surgical treatments such as liver resection and transplantation are the best curative local treatments for HCC 2. However, the rate of recurrence and metastasis are still high even after curative hepatectomy 3. The rate of recurrence of HCC in patients who underwent curative surgical or regional therapy is 75% at the fifth year 4, and the rate of recurrence is 86.5% for intrahepatic metastasis and 13.5% for extrahepatic metastasis 5. At present, serum biomarkers, such as alpha‐fetoprotein (AFP) and prothrombin induced by vitamin K absence II (PIVKA II), and many clinicopathological factors are used for prognostic markers of HCC 6, 7, but they are not adequate to predict survival or recurrence after curative hepatectomy 8. Hence, new biomarkers that are effective for predicting prognosis, recurrence, and metastasis in HCC are highly needed.

In a previous study, we identified fatty acid‐binding protein 5 (FABP5) as a protein that was highly expressed in human HCC tissues and cell lines compared with normal liver tissues and hepatocytes 9, 10. FABP5, also known as psoriasis‐associated fatty acid‐binding protein, epidermal, or cutaneous fatty acid‐binding protein (PA‐, E‐, or C‐FABP), is an isoform of the FABPs, which are small (~15 kDa) soluble intracellular lipid‐binding proteins that bind a variety of retinoids and long‐chain fatty acids 11, 12, 13. FABPs transport lipids to cellular compartments for the storage of lipid droplets, trafficking and membrane synthesis, and transcriptional regulation 14. FABP5 functions to enhance the transcriptional activity of the nuclear receptor peroxisome proliferator‐activated receptor *β*/*δ*; promotes cell migration, proliferation, and survival; and also exhibits pro‐oncogenic activities 15, 16, 17. FABP5 is overexpressed in many human cancers including prostate 18, 19, esophageal 20, squamous cell carcinoma 21 and breast cancer 22, 23. However, no reports have examined the clinicopathological significance and underlying molecular mechanisms of FABP5 in HCC.

In this study, we evaluated the correlation between the expression of FABP5 and malignant behavior of HCC in human HCC tissues and HCC cell lines.

## Materials and Methods

### Patients and specimens

Human liver tissues were obtained from 243 patients who underwent surgical resection of primary HCC between 1997 and 2006 at the Department of Gastroenterological Surgery I, Hokkaido University Hospital. Clinical characteristics of the patients are summarized in Table 1. This study was approved by the Institutional Review Board of the Hokkaido University, School of Advanced Medicine. Informed consent was obtained from each patient in accordance with the Ethics Committees Guidelines for our institution.

**Table 1:** Clinical characteristics of 243 HCC patients

Characteristics	
Age (years), median (range)	63 (35–82)
Male:female	200:43
Etiology, *n* (%)	
HBV	98 (40.3)
HCV	92 (37.9)
Both HBV and HCV	8 (3.3)
Non‐B and Non‐C	53 (21.8)
*Pathological factors*	
HCC	
Differentiation (well‐mod/por)	184/61
Tumor size (≤5 cm/>5 cm)	173/70
Single/multinodular	186/57
Noncancerous liver	
Cirrhosis/noncirrhosis	87/156

HBV, hepatitis B Virus; HCV, hepatitis C Virus; Non‐B Non‐C, without HBV and HCV; HCC, hepatocellular carcinoma; Well‐mod, well or moderately differentiated; Por, poorly differentiated.

### Immunohistochemical study

Formalin‐fixed and paraffin‐embedded specimens were cut by microtome and mounted on slides. Deparaffinization and antigen retrieval were performed, using PT Link and EnVision FLEX Target Retrieval Solution High pH (Dako, Glostrup, Denmark). Endogenous peroxidase activity was blocked using a peroxidase‐blocking solution. Sections were incubated with anti‐FABP5 primary antibody in a moist chamber. Sections were then incubated in labeled polymer (EnVision FLEX HRP; Dako). Staining was visualized, using 3,3′ diaminobenzidine, and samples were counterstained with hematoxylin and manually dehydrated and coverslipped. Antibody against FABP5 is listed in Table S3.

### Cell culture

Human HCC cell lines HLE, JHH5, HuH‐7, and HepG2 were purchased from the Japanese Collection of Research Bioresources Cell Bank (Osaka, Japan). Li‐7 cells were purchased from the RIKEN BioResource Center (Ibaraki, Japan). Hep3B cells were purchased from American Type Culture Collection. HLE, HuH‐7, HepG2, and Li‐7 cells were cultured in Dulbecco's Modified Eagle Medium supplemented with 10% fetal bovine serum (FBS). Hep3B cells were cultured in Eagle's Minimum Essential Medium supplemented with 10% FBS and JHH5 cells were cultured in Williams's medium with 10% FBS.

### Establishment of FABP5 overexpressing cells

The full‐length cDNA encoding human FABP5 was obtained from human whole blood by reverse transcription‐polymerase chain reaction (PCR). The FABP5 primer sequences are listed in Table S1. The cDNA was cloned into pLVSIN‐IRES‐ZsGreen1 vector (Takara Bio, Shiga, Japan) according to the manufacturer's instructions. Lentiviral supernatants were produced using the Lenti‐X HTX Packaging System (Clontech Laboratories, Mountain View, CA) and used for transduction of HCC cell lines. For the negative control, we transduced HCC cell lines with supernatants from empty vector cells. Fluorescence activated cell sorting was performed twice to select stable clones.

### Establishment of FABP5 knockdown cells

Lentiviral short hairpin RNA (shRNA) transduction particles targeting FABP5 and the negative control vector were purchased from Sigma‐Aldrich (St. Louis, MO) and transduced into HCC cell lines according to the manufacturer's instructions. Puromycin (2.5 *μ*g/mL) was used to select stable clones. The shRNA sequences are listed in Table S1.

### PCR array

Gene profiles were analyzed by the Human Cancer Pathway Finder RT2 Profiler PCR Array (Qiagen, Valencia, CA) according to the manufacturer's instructions. Genes showing more than 1.5‐fold change were considered biologically significant and selected for further analysis.

### Western blot analysis

Cell proteins were extracted, using the Subcellular Protein Fractionation Kit for Cultured Cells (Pierce Biotechnology, Waltham, MA). Samples were mixed with sodium dodecyl sulfate sample buffer (Bio‐Rad, Hercules, CA), boiled at 95°C for 5 min, separated by sodium dodecyl sulfate‐polyacrylamide gel electrophoresis, and transferred to polyvinylidene difluoride membranes (GE Healthcare, Little Chalfont, U.K.). Membranes were immunoblotted, using primary antibodies against FABP5, E‐cadherin, *N*‐cadherin, glyceraldehyde 3‐phosphate dehydrogenase (GAPDH), LaminB1, *β*‐catenin, SNAI1 (Snail) and ZO‐1 (see Table S3).

### Cell invasion and migration assay

Invasion and migration assays were performed in 24‐well Matrigel invasion chambers (8.0 *μ*m pore size; Corning, New York, NY) and polyester membrane inserts (8.0 *μ*m pore size; Corning) according to the manufacturer's instructions. Briefly, medium containing 10% FBS was added to lower chambers and 5 × 10^4^ cells in FBS‐free medium were seeded into upper chambers. After 48 h, cells on the upper chamber were removed with a cotton swab. Membranes were stained, using the Diff‐Quik staining kit (Sysmex, Hyogo, Japan) and cells were counted.

### Focus formation assay

A total of 1 × 10^4^ cells were plated in 60 mm dishes and the medium was changed twice per week. After 2 week, the cells were fixed and stained with the Diff‐Quik staining Kit. Colonies >2 mm in diameter were counted using an optical microscope. Three independent experiments were performed.

### Soft agar colony formation assay

Cells were plated at a density of 5 × 10^3^ cells in 60 mm dishes in growth medium containing 0.5% agar (2 mL per well) on top of a layer of growth medium containing 1.0% agar (2 mL per well). Culture medium (500 *μ*L) with 10% FBS was added on top of the agar. The cell suspension was plated and cultured in a 37°C incubator for 14 days. After 14 days, the number of colonies was counted. Three independent experiments were performed.

### Cell proliferation assay

Cells were trypsinized and seeded into 96‐well culture plates 24 h after transfection at a density of 1 × 10^3^ cells/well. The cells were harvested at different time points (0, 1, 2, 3, and 4 days) for growth assay using the 3‐(4,5‐dimethylthiazol‐2‐yl)‐5‐(3‐carboxymethoxyphenyl)‐2‐(4‐sulfophenyl)‐2H‐tetrazolium (MTS) kit (Cell Titer 96 AQ; Promega, Madison, WI) following the manufacturer's protocol, and the absorption was read at 490 nm. The data were presented as the mean fold increase ± SD.

### Tumor growth in xenograft

All animal experimental protocols were approved by the Ethics Review Committee for Animal Experimentation of Hokkaido University. HCC cells (1 × 10^6^ cells) were suspended in 50 *μ*l phosphate‐buffered saline and 50 *μ*L Matrigel (Corning) and injected subcutaneously into both shoulders of four‐wk‐old male BALB/cSlc‐nu/nu mice (Sankyo Labo Service Corporation, Tokyo, Japan). Three mice were used for each cell line. Tumor size (length and width) was measured every 4 days with a caliper and tumor *V* (mm^3^)  =  width [2] (mm^2^) × length (mm)/2. Tumor growth was observed for at least 20 days.

### Experimental metastasis model

Four‐week‐old male BALB/cSlc‐nu/nu mice in each experimental group were injected with each HCC cell line. To establish the liver metastasis model, 1 × 10^6^ HCC cells were injected intravenously through right branches of portal vein by laparotomy under anesthesia. The mice were sacrificed after 6–7 week and the numbers of surface liver metastases were counted. We also established a lung metastasis model by injecting 1 × 10^6^ HCC cells into the tail veins of nude mice. The mice were observed for long distance lung metastasis at 8 week, and then lungs were dissected. Each model was produced in a group involving three mice. The liver and lung tissues were fixed with 10% formalin and prepared for histological analysis by hematoxylin and eosin (H&E) staining.

### Statistical analysis

Data analysis was performed, using JMP 11 (SAS Institute, Cary, NC). The patient overall survival and relapse‐free survival rates were determined, using the Kaplan–Meier method and compared between groups by the log‐rank test. Univariate analysis was performed and selected variables were analyzed, using the Cox proportional hazard model for multivariate analysis. Categorical data were analyzed with Chi‐squared test. The differences between groups were determined by the Student *t*‐test. Statistical significance was defined as *P* < 0.05.

## Results

### Expression of FABP5 in HCC tissues

**Figure 1:**
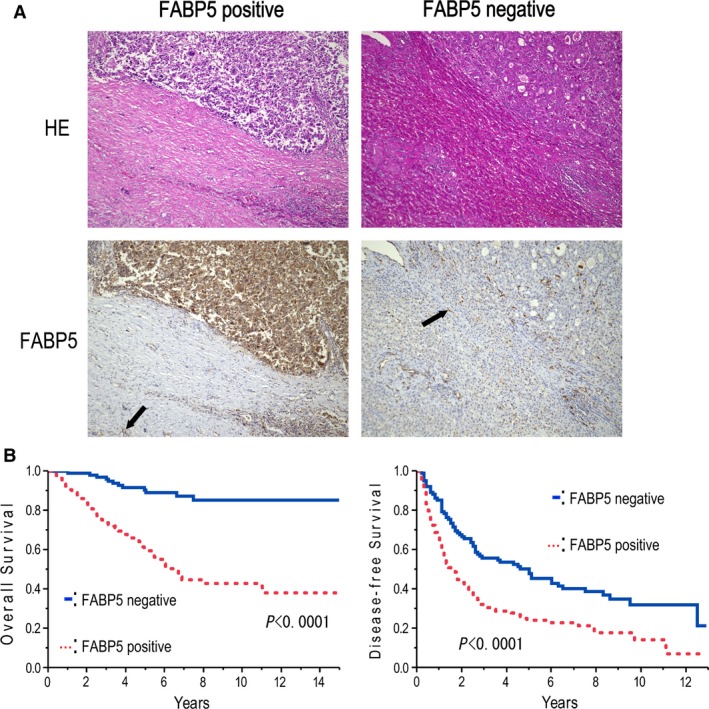
Expression of FABP5 in HCC. (A) Representative hematoxylin and eosin (H&E) staining and immunohistochemical staining of FABP5 in two pairs of HCC and adjacent nontumorous tissues (original magnification, 200×). Black arrow indicates staining of Kuppfer cell. (B) Correlation of FABP5 expression with poor overall survival and disease‐free survival rates of HCC patients. FABP5, fatty acid‐binding protein 5; HCC, hepatocellular carcinoma.

An immunohistochemical study was performed to examine the expression pattern of FABP5 in 243 paired HCC and adjacent noncancerous liver tissue samples, and the staining was separately evaluated by two independent pathologists. The intensity of immunohistochemical staining was scored according to the percentage of stained tumor cells stronger than the immunoreactivity of Kuppfer cells (0; <5%, 1; 5–20%, 2: 20–60%, 3; 60–100%). The FABP5 expression was also divided into a negative expression group (0–1) and a positive expression group (2–3). All normal liver cells were negatively stained. Representative images of two pairs of HCC and adjacent nontumorous tissues are shown in Figure 1A. Results showed that 57.2% (139/243) of HCC patients showed positive FABP5 staining. FABP5 positivity correlated with the histologic presence of microvascular invasion (*P *=* *0.0001), tumor differentiation (*P *=* *0.0337), tumor size (*P *=* *0.0001), tumor markers such as AFP (*P *=* *0.0022) and PIVKA‐II (*P *=* *0.0025) (Table 2). Patients with positive FABP5 staining showed a significantly poorer prognosis (5 year overall survival rate: 60.6% vs. 90.5% in patients with positive FABP5 staining versus patients with negative FABP5 staining, respectively; *P *<* *0.0001) and higher recurrence (5 year disease‐free survival rate: 24.2% vs. 47.9%, in patients with positive FABP5 staining vs. patients with negative FABP5 staining, respectively; *P *<* *0.0001) compared with FABP5 negative patients (Fig. 1B). Cox's multivariate proportional hazards model indicated that FABP5 expression was an independent predictor of survival (hazard ratio (HR): 5.276, 95% confidence interval (CI): 2.928–10.347, *P *<* *0.0001) and recurrence (HR: 1.952, 95% CI: 1.428–2.691, *P *<* *0.0001) in HCC patients after curative resection (Tables 3 and 4).

**Table 2:** Correlation between the expression of FABP5 and clinicopathological characteristics

	FABP5				FABP5		
	Positive	Negative	*P*		Positive	Negative	*P*
AFP				Differentiation			
>20 ng/mL	37	14	0.0022	Por	112	82	0.0337
≤20 ng/mL	94	97		Well‐mod	20	29	
PIVKA‐II				Microvascular invasion			
>40 mAU/mL	67	35	0.0025	Positive	32	6	0.0001
≤40 mAU/mL	65	76		Negative	100	105	
Tumor size				Tumor number			
>5 cm	96	53	0.0001	Multinodular	26	31	0.1315
≤5 cm	36	58		Single	106	80	

*P*‐values < 0.05 indicate statistical significance.

**Table 3:** Univariate and multivariate analysis associated with overall survival

Variable	HR	95% CI	*P*
Univariate analysis			
Age (>60 vs. ≤60 years)	1.319	0.791–2.234	0.291
Sex (male vs. female)	1.082	0.587–2.135	0.808
HBV (positive vs. negative)	0.784	0.430–1.427	0.426
HCV (positive vs. negative)	1.456	0.828–2.578	0.192
AFP (>20 vs. ≤20 ng/mL)	1.361	0.846–2.209	0.205
PIVKA‐II (>40 vs. ≤40 mAU/mL)	1.227	0.725–2.112	0.449
Tumor size (>5 vs. ≤5 cm)	1.159	0.613–2.332	0.659
Differentiation (por vs. well‐mod)	1.111	0.586–1.989	0.735
UICC stage (II‐IV vs. I)	1.275	0.486–3.747	0.631
Microvascular invasion (positive vs. negative)	2.607	1.474–4.503	***0.001***
Tumor number (multinodular vs. single)	2.187	1.209–3.875	***0.010***
FABP5 (positive vs. negative)	4.999	2.713–9.978	***<0.0001***
Multiivariate analysis			
*Overall survival*			
Microvascular invasion (positive vs. negative)	2.434	1.398–4.120	***0.0021***
Tumor number (multinodular vs. single)	2.490	1.454–4.172	***0.0012***
FABP5 (positive vs. negative)	5.276	2.928–10.347	***<0.0001***

HBV, hepatitis B Virus; HCV, hepatitis C Virus; AFP, alpha‐fetoprotein; PIVKA II, prothrombin induced by vitamin K absence II; UICC, UICC: Union for International Cancer Control; FABP5, fatty acid‐binding protein 5.

*P*‐values < 0.05 indicate statistical significance.

**Table 4:** Univariate and multivariate analysis associated with disease‐free survival

Variable	HR	95% CI	*P*
Univariate analysis			
Age (>60 vs. ≤60 years)	1.223	0.865–1.727	0.253
Sex (male vs. female)	1.053	0.679–1.583	0.811
HBV (positive vs. negative)	1.069	0.703–1.620	0.755
HCV (positive vs. negative)	1.218	0.820–1.819	0.330
AFP (>20 vs. ≤20 ng/mL)	1.142	0.832–1.568	0.411
PIVKA‐II (>40 vs. ≤40 mAU/mL)	1.032	0.738–1.447	0.854
Tumor size (>5 vs. ≤5 cm)	1.335	0.897–2.029	0.157
Differentiation (por vs. well‐mod)	1.323	0.886–2.040	0.175
UICC stage (II–IV vs. I)	1.386	0.892–2.094	0.142
Microvascular invasion (positive vs. negative)	1.148	0.652–2.073	0.636
Tumor number (multinodular vs. single)	1.975	1.356–2.834	***0.0005***
FABP5 (positive vs. negative)	1.702	1.214–2.403	***0.002***
Multiivariate analysis			
Tumor number (multinodular vs. single)	1.947	1.368–2.724	***0.0003***
FABP5 (positive vs. negative)	1.952	1.428–2.691	***<0.0001***

HBV, hepatitis B Virus; HCV, hepatitis C Virus; AFP, alpha‐fetoprotein; PIVKA II, prothrombin induced by vitamin K absence II; UICC, Union for International Cancer Control; FABP5, fatty acid‐binding protein 5.

### FABP5 modulates cell invasion and migration in HCC

**Figure 2:**
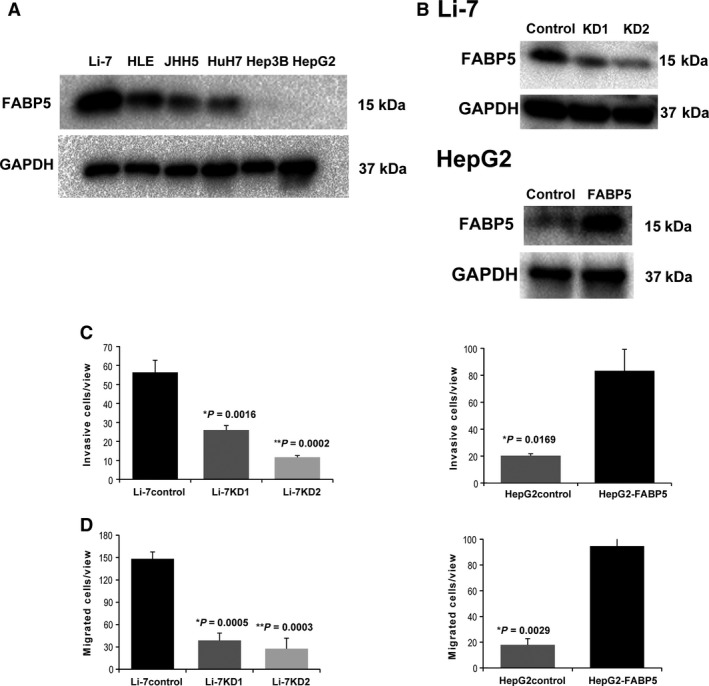
FABP5 promotes HCC cell invasion and migration. (A) Western blot analysis of FABP5 expression in HCC cell lines. (B) Top panel: Two shRNAs against FABP5 effectively downregulated FABP5 protein in Li‐7 as detected by western blot analysis. Bottom panel: Overexpression of FABP5 in HepG2 ectopically expressing FABP5. (C) Silencing FABP5 inhibited cell invasion in Li‐7, while promotion of cell invasion was observed by overexpression of FABP5 in HepG2. (D) Migration assays show that knockdown of FABP5 inhibited cell migration in Li‐7, whereas overexpression of FABP5 promoted cell migration in HepG2. The results are expressed as the mean ± SD of three independent experiments. FABP5, fatty acid‐binding protein 5; HCC, hepatocellular carcinoma.

We next examined the association of FABP5 expression with HCC malignant potential in HCC cell lines (Fig. 2A). Western blot analysis showed that the expression of FABP5 was high in invasive cell lines (Li‐7, HLE) and low in noninvasive cell lines (Hep3B, HepG2). We established stable knockdown Li‐7 cell lines, using two specific lentiviral shRNA vectors against FABP5 (Li‐7KD1 and Li‐7KD2) and a control Li‐7 cell line (Li‐7control). We also constructed a HepG2‐FABP5 cell line that overexpressed FABP5 and a HepG2 control cell line (HepG2control), using empty vector (Fig. 2B). Matrigel invasion assays demonstrated that knockdown of FABP5 significantly reduced invasiveness in Li‐7 cells (*P *<* *0.05) while overexpression of FABP5 in HepG2 cells resulted in stimulation of invasive capacity (*P *<* *0.05) (Fig. 2C). Migration assays also showed inhibition of migration by knockdown of FABP5 (*P *<* *0.05) and enhancement of migration by the overexpression of FABP5 (*P *<* *0.05) (Fig. 2D).

### FABP5 modulates HCC cell proliferation and colony formation

**Figure 3:**
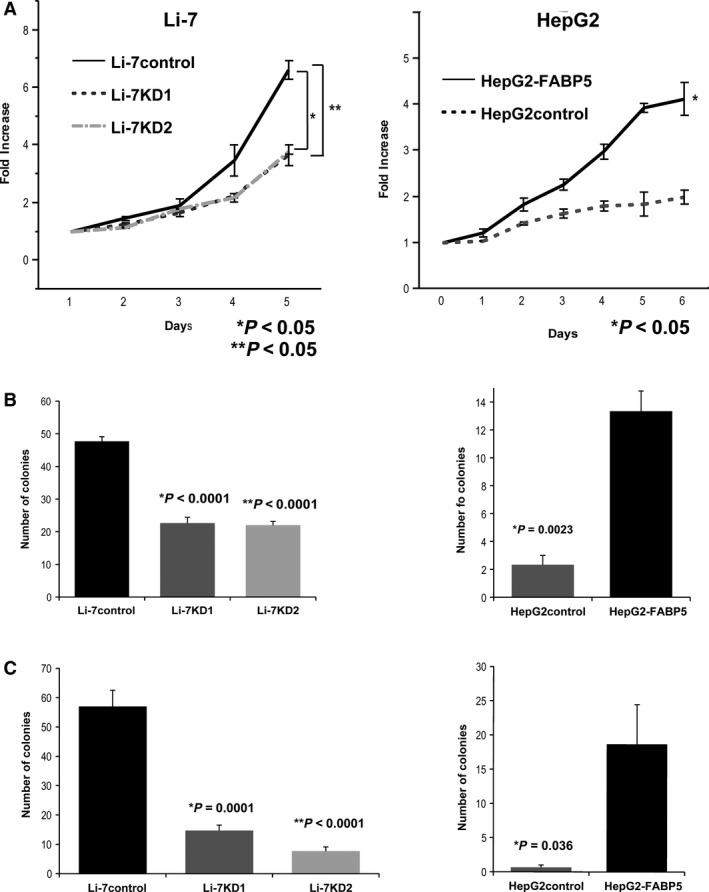
Fatty acid‐binding protein 5 (FABP5) promotes hepatocellular carcinoma cell proliferation and colony formation. (A) Rate of cell proliferation of Li‐7control, Li‐7KD1, and Li‐7KD2 cells (left), as well as HepG2 cells stably expressing FABP5 or empty vector (right) by MTS assay. The results are expressed as the mean ± SD of three independent experiments. (B) Representative bar charts of foci formation by indicated cells. Values are reflected as the mean ± SD of three independent experiments. (C) Li‐7control, Li‐7KD1, and Li‐7KD2 cells were subjected to soft agar colony formation assays. Values are reflected as the mean ± SD of three independent experiments.

MTS assay showed a significant inhibition in proliferation by downregulation of FABP5 expression in Li‐7 cells (*P *<* *0.05) and significantly increased proliferation by upregulation of FABP5 expression in HepG2 cells (*P *<* *0.05) (Fig. 3A). Colony formation assay showed significantly reduced colonies in the FABP5 downregulated Li‐7 cell line compared with the control cell line (*P *<* *0.05) (Fig. 3B). In contrast, upregulation of FABP5 resulted in an increase in colony formation in the HepG2 cell line (*P *<* *0.05) (Fig. 3B). Similarly, soft agar assay showed that knockdown of FABP5 significantly inhibited colony formation in soft agar in Li‐7 cells compared with control cells, while overexpression of FABP5 in HepG2 cells resulted in an increase in colonies (*P *<* *0.05) (Fig. 3C).

### FABP5 promotes tumor development in BALB/c nude mice

**Figure 4:**
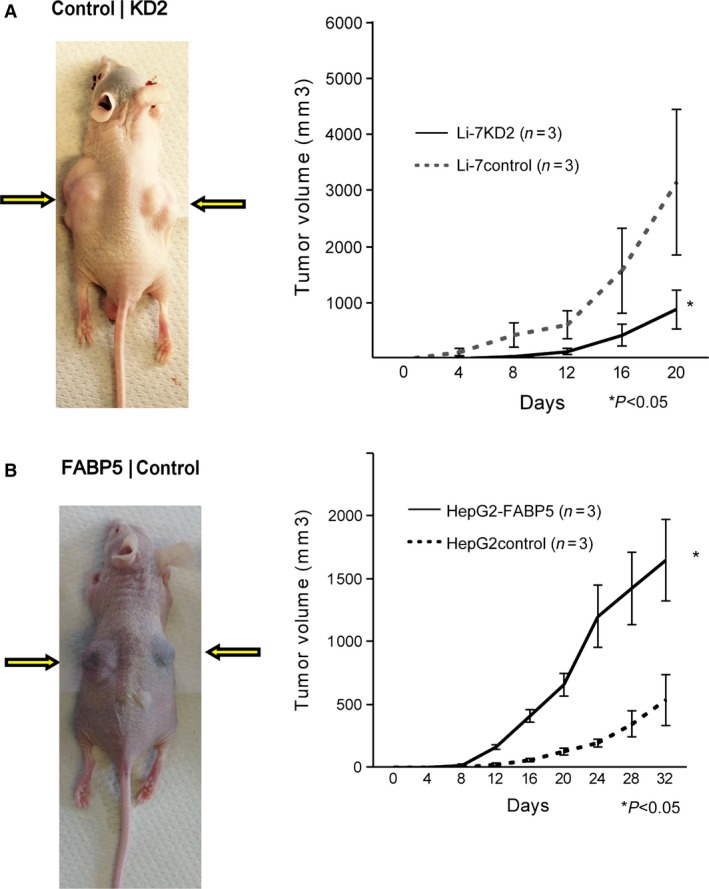
FABP5 promotes tumor growth. (A) Li‐7control (control) and Li‐7KD2 (KD2) cells were subcutaneously injected to left and right shoulder, respectively. Growth curve of subcutaneous tumors from Li‐7control and Li‐7KD2 cells is shown on the right. (B) Images of xenograft tumors injected with HepG2‐FABP5 (FABP5) and HepG2control (control) cells. Graph of tumor volume of subcutaneous tumors from HepG2control and HepG2‐FABP5 cells is shown on the right. FABP5, fatty acid‐binding protein 5. Values are reflected as the mean ± SD of three independent experiments. *P‐values <0.05 indicate statistical significance.

We found that the volume of tumors developed from FABP5 high‐expressing cells (Li‐7 control or HepG2‐FABP5) was significantly larger than that of tumors from FABP5 low‐expressing cells (Li‐7KD2 or HepG2 control) (*P *<* *0.05) (Fig. 4A and B).

### FABP5 enhances metastatic potential of HCC cell lines in vivo

**Figure 5:**
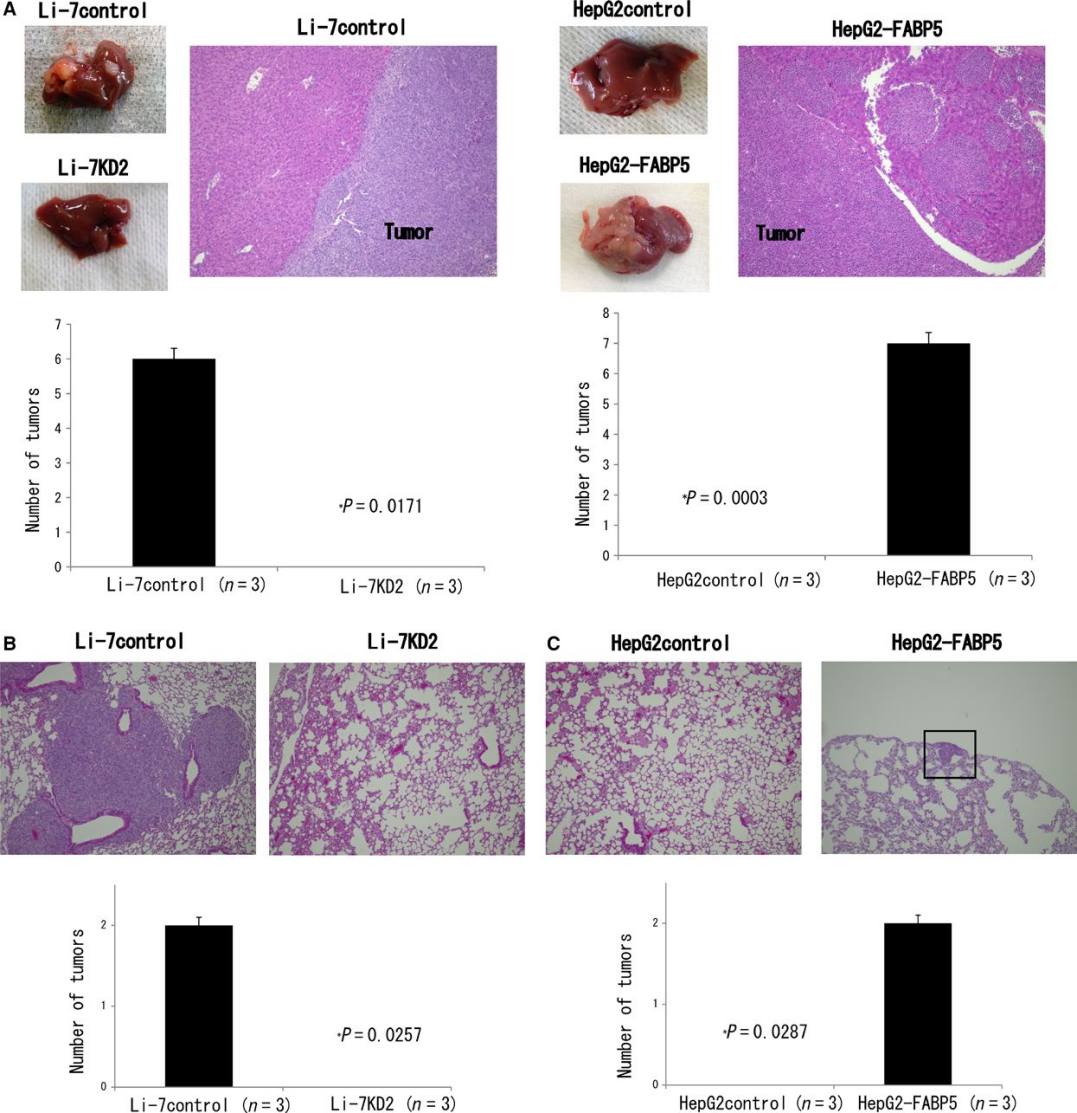
FABP5 promotes hepatocellular carcinoma metastasis. (A) Images of liver metastasis and H&E staining from Li‐7control and HepG2‐FABP5 cells. No apparent liver metastases were observed in Li‐7KD2 and HepG2control groups. (B) H&E staining of lung tissues from Li‐7control and Li‐7KD2 cells. (C) Representative H&E staining of lung tissues from HepG2control and HepG2‐FABP5 cells. Black lesion indicates lung metastatic lesion. FABP5, fatty acid‐binding protein 5.

In the liver metastasis model, we observed metastatic nodules in the FABP5 high expression group in contrast to no visible nodules in FABP5 low expression group. Histological analysis by H&E staining also confirmed the surface nodules as metastatic tumors (Fig. 5A and B). In the lung metastasis model, microscopic lung metastases were only observed in the Li‐7 control and HepG2‐FABP5 groups (Fig. 5C and D).

### FABP5 induces epithelial‐to‐mesenchymal transition

We next performed a human cancer pathway finder PCR array, using HepG2‐FABP5 and HepG2 control cells to examine the hypothesis that FABP5 affects the expression of genes that contribute to HCC aggressiveness. The results revealed upregulated mRNA expression of the epithelial‐to‐mesenchymal transition (EMT)‐related gene, Snail, in HepG2‐FABP5 cells compared with HepG2 controls (Table S2).

**Figure 6:**
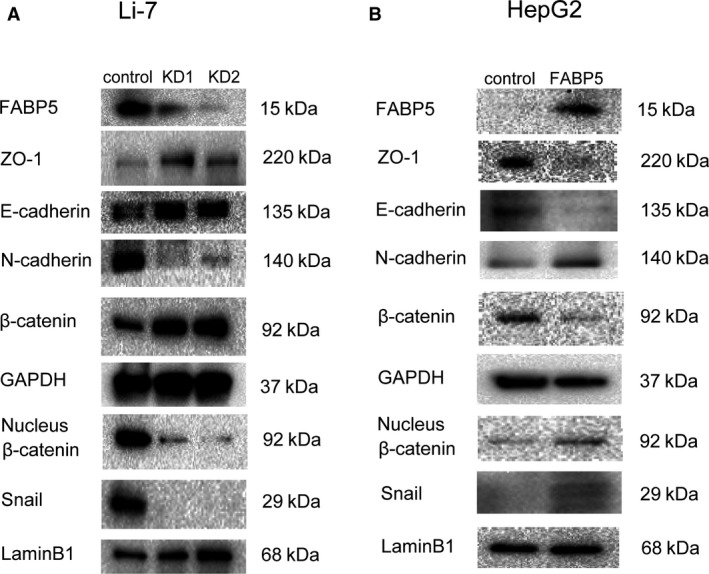
FABP5 activates Snail to induce epithelial‐to‐mesenchymal transition. (A) Western blot analysis of FABP5, ZO‐1, E‐cadherin, N‐cadherin, *β*‐catenin, nucleus *β*‐catenin, and Snail expressions in Li‐7control, Li‐7KD1, and Li‐7 KD2cells. GAPDH and LaminB1 were used as the loading controls. (B) Protein levels of FABP5, ZO‐1, E‐cadherin, N‐cadherin, *β*‐catenin, nucleus *β*‐catenin, and Snail in HepG2control and HepG2‐FABP5 cells. GAPDH and LaminB1 were used as the loading controls. FABP5, fatty acid‐binding protein 5; GAPDH, Glyceraldehyde 3‐phosphate dehydrogenase.

To explore the effect of FABP5 on EMT, western blot analysis was performed to investigate the expression levels of epithelial (E‐cadherin and ZO‐1) and mesenchymal (N‐cadherin) markers. Expression levels of E‐cadherin and ZO‐1 were significantly increased by knockdown of FABP5 while N‐cadherin was decreased in Li‐7 cells and Snail expression and nuclear translocation of *β*‐catenin were reduced (Fig. 6A). We observed the opposite effects in HepG2 cells upregulated for FABP5 (Fig. 6B). Immunohistochemical assay of human HCC tissue was also revealed the correlation between high expression of FABP5 and high expression of N‐cadherin or low expression of E‐cadherin (Fig. S1A). A similar correlation of the expression of N‐cadherin, E‐cadherin with the expression of FABP5 was also observed in mouse liver metastatic tissues (Fig. S1B).

## Discussion

This is the first report describing the clinical significance of positive expression FABP5 in human HCC and its correlation with malignant behavior, recurrence and prognosis. We revealed a significant role of FABP5 in tumor progression, invasion and metastasis of HCC through the induction of EMT.

In this study, we found that FABP5 was frequently overexpressed in human HCC tissues compared with the paired noncancerous tissues. Furthermore, FABP5 overexpression was correlated with tumor size, vascular invasion, poor differentiation, advanced UICC stage, and distant metastasis. HCC patients with positive FABP5 expression had worse prognoses and higher recurrence rates than patients with negative FABP5 expression. In addition, multivariate analysis revealed that high expression of FABP5 was an independent and significant risk factor for survival and recurrence. These data strongly indicate that FABP5 contributes to the tumor progression, invasion, and metastasis of HCC.

Our study demonstrated that overexpression of FABP5 promoted cell proliferation, invasion, and migration in vitro and also promoted tumor growth in mice models. Furthermore, knockdown of FABP5 showed marked inhibition of malignant abilities both in vitro and in vivo. Moreover, the role of FABP5 in promoting tumor metastasis was supported by the in vivo experimental metastasis assay, and reverse effects were observed upon knockdown of FABP5. Together these results suggest that FABP5 might play a crucial role in tumor progression, invasion and metastasis in HCC.

Metastasis is defined as the spread of cancer cells from the initial or primary site of disease to another part of the body. The stages of metastasis include the detachment of tumor cells from the primary tumor, invasion into surrounding tissue, intravasation into blood or lymphatic vessels, dissemination in the blood stream or the lymphatic system, and finally extravasation and outgrowth at a secondary site 24. The key process of cancer cell migration, invasion and metastatic dissemination is called EMT, a multi‐step morphogenetic process during which epithelial cells loss their epithelial properties accompanied by total acquisition of mesenchymal characteristics. The important molecular feature of EMT is the downregulation of E‐cadherin 25, 26. Degradation of cell‐to‐cell adhesion by disrupting the E‐cadherin axis is a major event in the transition from epithelial cells to mesenchyme 27. Until now, no reports have examined the correlation of FABP5 and EMT. In this study, we investigated whether FABP5 causes tumor progression and metastasis by way of induction of the EMT pathway. Moreover, no reports have analyzed a molecular pathway for cancer metastasis in connection with EMT. Our data showed that overexpression of FABP5 may have an effect in promoting EMT, as indicated by decreased protein expression of epithelial markers (E‐cadherin and ZO‐1) and increased expression of mesenchymal markers such as N‐cadherin. Furthermore, knockdown of FABP5 had the opposite effect. In addition, the expression level of Snail, a major transcription factor in EMT, was increased by FABP5 overexpression and decreased by downregulation of FABP5. The expression level of FABP5 correlated with the expression level of epithelial (E‐cadherin and ZO‐1) and mesenchymal (N‐cadherin) markers as well as Snail. Therefore, FABP5 activation might facilitate the increased invasiveness and metastasis of HCC through the induction of EMT.

Previous reports showed that high expression of FABP5 correlated with cancer metastasis, invasion and worse prognosis in various cancers 28, 29, 30. Other studies showed that overexpression of FABP5 promotes cancer metastasis by upregulating matrix metalloproteinase 9 (MMP‐9), a proteolytic enzyme that accelerates tumorigenesis and metastasis, and vascular endothelial growth factor (VEGF), one of the major proteins involved in tumor angiogenesis 28, 31. Moreover, previous studies have suggested that VEGF plays an important role of EMT through the regulation of Snail, and MMP‐9 promotes cytoskeleton remolding, destruction of basement membrane and also angiogenesis to assist tumor metastasis or invasion via EMT pathway 32, 33. These reports might also support the correlation of FABP5 and EMT on the point of tumor invasion and metastasis, which was certificated in this study.

Most HCC cases arise from patients with chronic liver diseases or cirrhosis. Hepatitis B virus (HBV) or hepatitis C virus (HCV) infections and alcoholic abuse were previously considered major risk factors of chronic hepatitis or cirrhosis 34, 35. However, owing to worldwide spread of immunization against HBV and antiviral therapies for HBV and HCV 36, 37, 38, nonalcoholic fatty liver disease (NAFLD) and its more aggressive form nonalcoholic steatohepatitis have recently emerged as higher risks of HCC. Recent studies reported that obesity, metabolic syndrome, and type 2 diabetes mellitus are significant risk factors for HCC and are associated with an increased risk of NAFLD 39, 40. Recently, free fatty acid accumulation has been reported as a risk factor of NAFLD, HCV‐related hepatocellular steatosis, and also HBV‐related HCC 41, 42, 43. Because FABPs bind a variety of retinoids and long‐chain fatty acids, inhibition of FABP5 might be a useful target for the prevention of metabolic hepatocarcinogenesis and therapy for highly malignant HCCs. However, we did not investigate the correlation between free fatty acid expression and FABP5 expression in HCC tissues in in vitro and in vivo studies using FABP5 inhibitors. Further examination of FABP5 functions is required to uncover the metabolic mechanisms of HCC pathogenesis, as well as to help develop potential clinical applications of metabolic therapy for HCC.

In conclusion, here we provide a novel insight into the significance of FABP5 expression on malignant behavior, recurrence and prognosis in HCC. Our findings suggest that FABP5 could play a crucial role of tumor progression, invasion and metastasis in HCC through EMT induction. FABP5 may serve as a valuable biomarker and potential molecular target for the development of HCC therapies.

## Conflict of Interest

None declared.

## Supporting information


**Table S1.** Primers and oligonucleotides.
**Table S2.** PCR array results.
**Table S3.** List of antibodies.

**Figure S1.** Correlation between expression of fatty acid‐binding protein 5 (FABP5) and N‐cadherin or E‐cadherin. (A) Immunohistochemical staining of N‐cadherin and E‐cadherin in high FABP5 human hepatocellular carcinoma tissue (original magnification, 200×). (B) Immunohistochemical staining of N‐cadherin and E‐cadherin in mouse metastatic liver tissues (original magnification, 200×).
