# Supplementary material for: Fatty acid‐binding protein 5 function in hepatocellular carcinoma through induction of epithelial–mesenchymal transition
Source: Cancer Med. 2017 Apr 4;6(5):1049–61. doi: 10.1002/cam4.1020 (PMC5430096; doi:10.1002/cam4.1020)
Supplement: Supplementary file 1 — Table S1. Primers and oligonucleotides. Table S2. PCR array results. Table S3. List of antibodies. [file CAM4-6-1049-s001.docx]

Table 1. Primers and oligonucleotides

| Name | Sequence |
| --- | --- |
| FABP5 primers |  |
| FABP5 F | GGTCCGCGGCCGCATGGCCACAGTTCAGCAG |
| FABP5 R | CCTGGGCGGCCGCTTATTCTACTTTTTCATAGATCCG |
| shRNA target sequences |  |
| FABP5 KD1 | CCGGGTATCATCACTTGTGATGGTACTCGAGTACCATCACAAGTGATGATACTTTTTG |
| FABP5 KD2 | CCGGGATGAATACATGAAGGAGCTACTCGAGTAGCTCCTTCATGTATTCATCTTTTTG |

FABP5: fatty acid binding protein 5

Table 2. PCR Array Results

| Function | Symbol | Name of Gene | Refseq | Fold Change |
| --- | --- | --- | --- | --- |
| Angiogenesis |  |  |  |  |
|  | PGF | Placental growth factor | NM_002632 | 9.3515 |
|  | TEK | TEK tyrosine kinase, endothelial | NM_000459 | 590.4356 |
|  | VEGFC | Vascular endothelial growth factor C | NM_005429 | 1.6957 |
| EMT |  |  |  |  |
|  | SNAI1 | Snail homolog 1 (Drosophila) | NM_005985 | 10.4451 |
|  | SOX10 | SRY (sex determining region Y)-box 10 | NM_006941 | 297.9553 |
| Apoptosis |  |  |  |  |
|  | FASLG | Fas ligand (TNF superfamily, member 6) | NM_000639 | 15.2096 |

EMT: epithelial–to-mesenchymal transition

TNF: tumor necrosis factor

Table 3. List of antibodies

| Target | species | Dilution | Vendor |
| --- | --- | --- | --- |
| FABP5 | Rabbit | 1:750 | Abcam |
| ZO-1 | Rabbit | 1:500 | Cell Signaling |
| E-cadherin | Rabbit | 1:200 | Cell Signaling |
| N-cadherin | Rabbit | 1:200 | Cell Signaling |
| β-catenin | Rabbit | 1:1000 | Cell Signaling |
| GAPDH | Rabbit | 1:1000 | Cell Signaling |
| Snail | Rabbit | 1:500 | Cell Signaling |
| LaminB1 | Rabbit | 1:500 | Cell Signaling |
