# Supplementary material for: Fatty acid‐binding protein 5 function in hepatocellular carcinoma through induction of epithelial–mesenchymal transition
Source: Cancer Med. 2017 Apr 4;6(5):1049–61. doi: 10.1002/cam4.1020 (PMC5430096; doi:10.1002/cam4.1020)
Supplement: Supplementary file 2 — Figure S1. Correlation between expression of fatty acid‐binding protein 5 (FABP5) and N‐cadherin or E‐cadherin. (A) Immunohistochemical staining of N‐cadherin and E‐cadherin in high FABP5 human hepatocellular carcinoma tissue (original magnification, 200×). (B) Immunohistochemical staining of N‐cadherin and E‐cadherin in mouse metastatic liver tissues (original magnification, 200×). [file CAM4-6-1049-s002.docx]

**Supplementary Fig.1** Correlation between expression of FABP5 and N-cadherin or E-cadherin. (A) Immunohistochemical staining of N-cadherin and E-cadherin in high FABP5 human HCC tissue (original magnification, 200×). (B) Immunohistochemical staining of N-cadherin and E-cadherin in mouse metastatic liver tissues (original magnification, 200×).
